# Nsd2 Represses Endogenous Retrovirus MERVL in Embryonic Stem Cells

**DOI:** 10.1155/2021/6663960

**Published:** 2021-01-16

**Authors:** Tingting Gao, Fuquan Chen, Wenying Zhang, Xuan Zhao, Xiao Hu, Xinyi Lu

**Affiliations:** State Key Laboratory of Medicinal Chemical Biology, College of Pharmacy, Nankai University, Tianjin 300350, China

## Abstract

The facilitates chromatin transcription (FACT) complex is a histone H2A/H2B chaperone, which represses endogenous retroviruses (ERVs) and transcription of ERV-chimeric transcripts. It binds to both transcription start site and gene body region. Here, we investigated the downstream targets of FACT complex to identify the potential regulators of MERVL, which is a key 2-cell marker gene. H3K36me2 profile was positively correlated with that of FACT component Ssrp1. Among H3K36me2 deposition enzymes, *Nsd2* was downregulated after the loss of *Ssrp1*. Furthermore, we demonstrated that Nsd2 repressed the expression of ERVs without affecting the expression of pluripotency genes. The expression of MERVL and 2-cell genes was partially rescued by *Nsd2* overexpression. The enrichment of H3K36me2 decreased on MERVL-chimeric gene in ESCs without *Ssrp1*. Our study discovers that Nsd2 is a repressor of MERVL, and FACT partially represses MERVL expression by regulating the expression of *Nsd2* and its downstream H3K36me2.

## 1. Introduction

Endogenous retroviruses are important components of the mammalian genome [1]. They are usually silenced by host cells to maintain genome stability. However, studies also show that ERVs are functional during development and in mouse embryonic stem cells (ESCs) [2–7]. For example, MERVL marks the 2-cell (2C) embryos and a minority of 2C-like cells within the ESC population [8, 9]. MERVL can be silenced by various epigenetic regulators, such as histone H3 variants, H3K9 methyltransferases, and histone chaperones [10–15]. Recently, we found that H2A/H2B histone chaperone FACT (facilitates chromatin transcription) complex participated in the repression of MERVL and MERVL-derived cryptic transcripts in ESCs [16]. FACT functions partially through Usp7 to remove H2Bub on MERVL and MERVL-fused genes [16]. However, the impact of *Usp7* depletion on MERVL induction is weaker than the loss of FACT complex itself [16]. This implies that there are other ways present for FACT complex to repress the expression of MERVL and its chimeric transcripts. Therefore, in this study, we aim to identify indirect pathways downstream of FACT complex in repressing the expression of MERVL.

We and others previously found that Ssrp1 binding was enriched around transcription start sites and on gene body region [16, 17]. Gene body region can be marked by H3K36me3 and H3K36me2 [18, 19]. An important H3K36 methyltransferase family is Nsd family. Here, we examine the role of Nsd family members at the downstream of FACT complex in repressing the ERV expression.

## 2. Methods

### 2.1. Cell Culture

E14 mouse embryonic stem cells (ESCs) were cultured on plates coated with sterile 0.1-0.2% gelatin (G1890, Sigma) in medium containing Dulbecco's modified Eagle's medium (Hyclone), 15% fetal bovine serum (Hyclone), 10 ng/ml leukaemia inhibitory factor (LIF) (Z03077, GenScript), 1% penicillin/streptomycin (P1400, Solarbio), 2 mM L-glutamine (Gibco), 0.1 mM nonessential amino acids (Gibco), and 0.1 mM *β*-mercaptoethanol (Sigma). ESCs were passaged every two days for maintenance.

### 2.2. Analysis of ChIP-seq Data

For ChIP-seq data analysis, all reads were first processed with Cutadapt to trim adaptor sequences and low-quality reads and subsequently mapped to the mouse mm10 genome assembly using Bowtie2. The correlation coefficient between Ssrp1, H3K4me3, H3K36me2, and H3K36me3 was determined by plotCorrelation from Deeptools. The ChIP-seq signal enrichment file was obtained by bamCompare from Deeptools, and the ChIP signal line plot was also generated by Deeptools. Gene structure information was inferred from Gencode.vM21 annotation file.

### 2.3. Reverse Transcription and qPCR

Total RNA was isolated from cells by RNAiso Reagent (B9109, Takara) in DEPC water (B501005, Sangon Biotech) following by DNase I treatment in RNase-free tubes (401001, NEST Biotechnology). Reverse transcription was performed with 1 *μ*g purified RNA using Transcriptor First Strand cDNA Synthesis Kit (4897030001, Roche) as described previously [20]. qPCR analysis was carried out using SYBR Green qPCR Master Mix (H97410, Yeasen) and a qPCR detection system (CFX384 Real-Time System, Bio-Rad) according to standard protocols. Primers are synthesized by Sangon Biotech and included in Table 1.

### 2.4. shRNA-Mediated Gene Depletion

The shRNAs targeting *Nsd2* were designed by an online tool (http://sirna.wi.mit.edu/) [21]. The targeting sequences of shRNAs are CCTGGTGCTCATGATACTAAA for shRNA1 and GAGCTGACTTTCAACTATAA for shRNA2. The shRNAs were synthesized by GENEWIZ corporation and cloned into pSuper-puro. 1 *μ*g plasmid was transfected into mouse ESCs with Polyjet (SignaGen). The cells were further cultured for three days under puromycin selection (1 *μ*g/ml) and harvested for RNA extraction.

### 2.5. Chromatin Immunoprecipitation (ChIP) Coupled qPCR

ChIP-qPCR was performed as described before [16]. Briefly, ESCs were harvested and crosslinked with 1% formaldehyde, and cell fixation was ceased with the addition of glycine. The cells were primarily lysed, and chromatin extracts were collected and sonicated for obtaining soluble chromatin fragments. The chromatin samples were incubated with specific antibody and immunoprecipitated on protein G magnetic beads (GenScript, the USA). The immunoprecipitated DNA was next eluted, decrosslinked, and analyzed by qPCR. For immunoprecipitation, the antibody used was anti-H3K36me2 (ab9049, Abcam).

### 2.6. Establishment of ESC Cell Lines Overexpressing Nsd2

Mouse *Nsd2* coding region was cloned into pCAG-3HA vector with hygromycin resistance and purified with a kit (1211-01, Biomiga). *Ssrp1*^−/−^ ESCs were transfected with 1 *μ*g plasmid expressing *Nsd2* via Polyjet reagent (SL100688, SignaGen) following the manufacturer's recommended protocol. The ESCs were continuously selected with 800 *μ*g/ml hygromycin B for 14 days. After selective cell culture, ESCs were collected for downstream experiments.

## 3. Results

### 3.1. FACT Complex Binding Is Correlated with H3K36 Methylation

**Figure 1:**
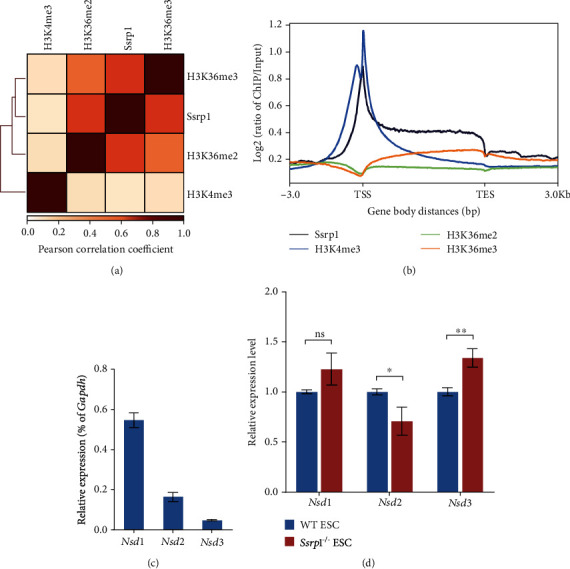
Nsd2-mediated H3K36me2 is correlated with Ssrp1 binding. (a) Genomic distribution profile of H3K36me2, H3K36me3, H3K4me3, and Ssrp1. Color scale represents the strength of Pearson's correlation. (b) ChIP-seq signal density enrichment of Ssrp1, H3K4me3, H3K36me2, and H3K36me3 (*Y*-axis) on gene body from TSS to TES (*X*-axis). H3K36me3 enrichment increased from TSS to TES. (c) Relative mRNA expression of *Nsd1*, *Nsd2*, and *Nsd3* in ESCs according to qPCR. (d) qPCR analysis of *Nsd1*, *Nsd2*, and *Nsd3* expression levels in WT ESC and *Ssrp1*^−/−^ ESC; data are shown as mean ± s.e.m.; *n* = 3 biologically independent repeats. ns: nonsignificant; ^∗^*p* < 0.05; ^∗∗^*p* < 0.01.

Previously, we found that FACT complex interacted with both promoter and gene body regions, which are marked by H3K36me2/3. Interestingly, the genomic distribution profile of H3K36me2 and H3K36me3 was positively correlated with that of Ssrp1 (Figure 1(a)), in contrast with the lower correlation strength of Ssrp1 with H3K4me3 (Figure 1(a)). Moreover, it was noteworthy that H3K36me3 enrichment on the gene body continuously increased from transcription start site (TSS) to transcription end site (TES) whereas the H3K36me2 was preferentially associated with TSS region and gradually decayed from TSS to TES (Figure 1(b)). The distribution profile of H3K36me2 was more similar to that of FACT complex than H3K36me3 (Figures 1(a) and 1(b)). Therefore, we further examined the expression of Nsd family genes (*Nsd1*, *Nsd2*, and *Nsd3*), which are known to mediate H3K36 methylation. *Nsd1* was expressed highest in ESCs while the expression of *Nsd2* and *Nsd3* was lower (Figure 1(c)). The expression of *Nsd1* and *Nsd3* remained unchanged or slightly upregulated in ESCs without FACT complex (Figure 1(d)). However, the *Nsd2* expression was downregulated in *Ssrp1*^−/−^ ESCs (Figure 1(d)), implying *Nsd2* as a potential downstream target gene of FACT complex.

### 3.2. Nsd2 Represses MERVL in ESCs

**Figure 2:**
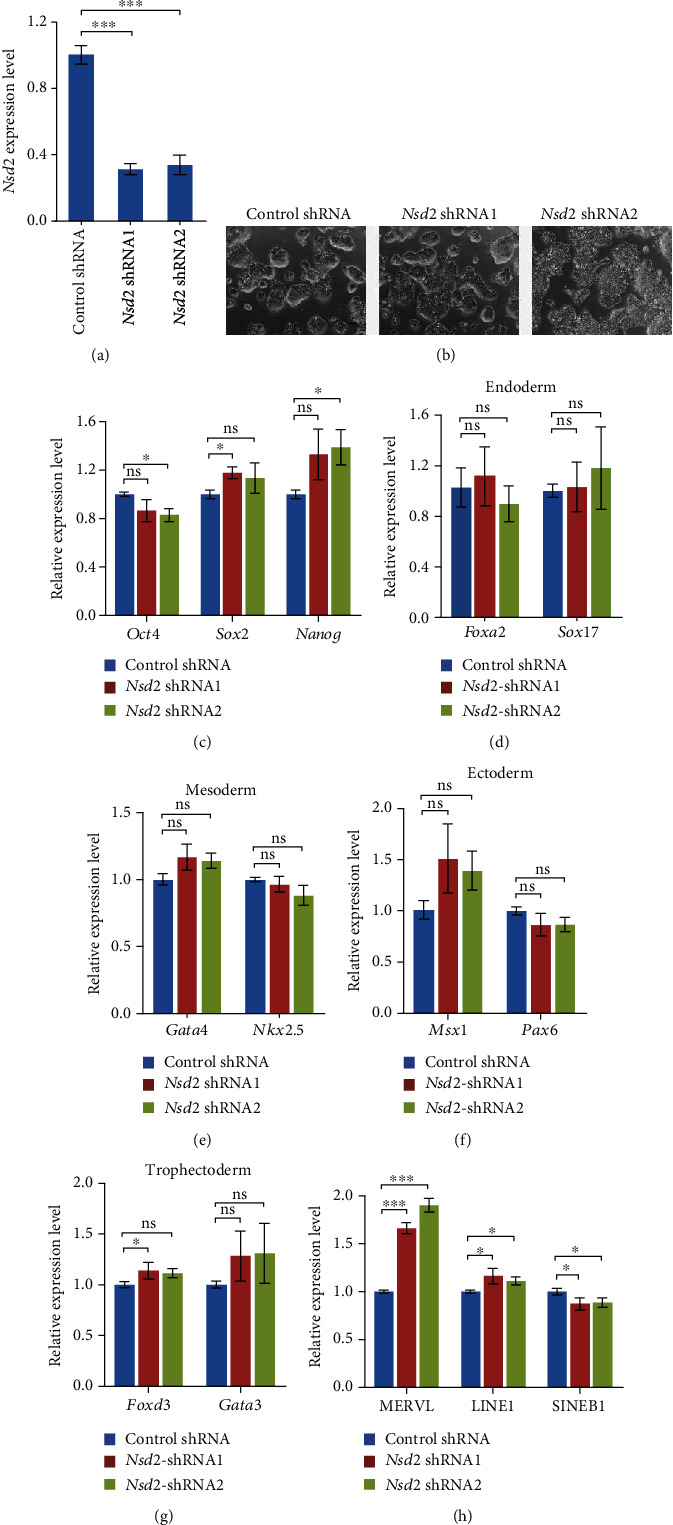
Depletion of *Nsd2* in ESCs. (a) qPCR analysis of the relative expression levels of *Nsd2* after *Nsd2* depletion in ESCs with normalization to *Gapdh*. Data were plotted as mean ± s.e.m., *n* = 3 biological repeats. (b) Cell morphology of *Nsd2* depletion in ESCs. Scale bar, 100 *μ*m. (c) qPCR analysis of pluripotent genes (*Oct4*, *Sox2*, and *Nanog*) in ESCs with *Nsd2* depleted. (d–g) qPCR analysis of endoderm markers *Foxa2* and *Sox17* (d), mesoderm markers *Gata4* and *Nkx2.5* (e), ectoderm markers *Msx1* and *Pax6* (f), and trophectoderm markers *Foxd3* and *Gata3* (g). (h) qPCR analysis of the expression levels of MERVL and other retrotransposons (LINE1, SINE B1) after *Nsd2* depletion in ESCs. The results were normalized to *Gapdh*. Data were shown as mean ± s.e.m.; *n* = 3 biologically independent replicates; ns: nonsignificant; ^∗^*p* < 0.05; ^∗∗^*p* < 0.01; ^∗∗∗^*p* < 0.001.

In agreement with the close resemblance of Ssrp1 binding profile and H3K36me2, the main chromatin-regulatory activity of Nsd2 is mediating the dimethylation of histone H3 at lysine 36 (H3K36me2) [19]. Hence, we depleted *Nsd2* in ESCs with two independent shRNAs to examine whether Nsd2 can regulate the expression of ERVs (Figure 2(a)). The depletion of *Nsd2* did not affect the cell morphology of ESCs (Figure 2(b)). Also, the expression of pluripotency genes (*Oct4*, *Sox2*, *Nanog*) was not disturbed by two *Nsd2* shRNAs at the same time (Figure 2(c)). The suppression of *Nsd2* by two independent shRNAs did not disrupt the expression of differentiation markers for endoderm (*Foxa2* and *Sox17*), mesoderm (*Gata4* and *Nkx2.5*), ectoderm (*Msx1* and *Pax6*), and trophectoderm (*Foxd3* and *Gata3*) at the same time (Figures 2(d)–2(g)), suggesting that ESCs remain undifferentiated without *Nsd2*. Intriguingly, the expression of MERVL was activated to ~2 folds by *Nsd2* depletion (Figure 2(h)), but other retrotransposons (LINE1 or SINE B1) were less activated or downregulated, confirming that Nsd2 acts downstream of FACT complex to repress the ERV expression. These results suggest that Nsd2 represses the expression of MERVL without affecting ESC pluripotency.

### 3.3. *Nsd2* Overexpression Rescues MERVL Expression in *Ssrp1*^−/−^ ESCs

**Figure 3:**
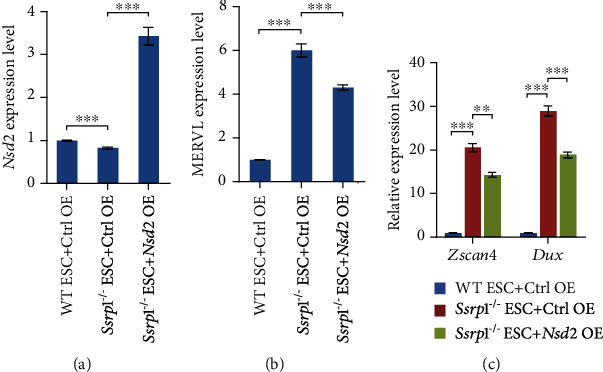
*Nsd2* overexpression rescues the expression of MERVL and 2-cell genes. (a) qPCR analysis of *Nsd2* expression after overexpression of *Nsd2* in *Ssrp1*^−/−^ ESC. The results were shown as mean ± s.e.m., *n* = 3 biologically independent replicates and normalized to *Gapdh*. (b) qPCR analysis of the expression of MERVL after overexpression of *Nsd2* in *Ssrp1*^−/−^ ESC. Mean ± s.e.m., *n* = 3. (c) qPCR analysis of the expression of 2-cell marker genes *Zscan4* and *Dux* after overexpression of *Nsd2* in *Ssrp1*^−/−^ ESC. Ctrl: control; OE: overexpression; mean ± s.e.m., *n* = 3; ^∗∗^*p* < 0.01; ^∗∗∗^*p* < 0.001.

Since Nsd2 represses the MERVL expression and *Nsd2* is downregulated in *Ssrp1*^−/−^ ESCs, we next asked whether restoration of the *Nsd2* expression in *Ssrp1*^−/−^ ESCs could rescue the expression of MERVL. Hence, we established an *Ssrp1*^−/−^ ESC line with *Nsd2* overexpressed. Our qPCR results showed that *Nsd2* was successfully overexpressed in *Ssrp1*^−/−^ ESCs (Figure 3(a)). Moreover, overexpression of *Nsd2* could partially reduce the expression of MERVL in *Ssrp1*^−/−^ ESCs (Figure 3(b)). Furthermore, the expression of 2-cell marker genes (*Zscan4* and *Dux*) was partially restored (Figure 3(c)). These results suggest that *Nsd2* is an important downstream target gene of Ssrp1 in repressing ERVs and 2-cell genes.

### 3.4. Nsd2-Mediated H3K36me2 Is Reduced on MERVL-Fused Genes in *Ssrp1*^−/−^ ESCs

**Figure 4:**
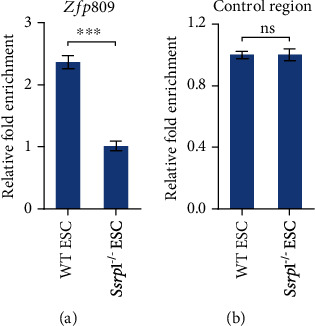
H3K36me2 enrichment on MERVL-fused genes. (a, b) ChIP-qPCR analysis of H3K36me2 enrichment on MERVL-fused gene *Zfp809* (a) and control region (b) in WT ESC and *Ssrp1*^−/−^ ESC with normalization to *Gapdh* and input; data shown as mean ± s.e.m. (*n* = 3 extracts). ns: nonsignificant; ^∗∗∗^*p* < 0.001.

We further investigated whether the target of Nsd2, H3K36me2, was affected at MERVL-fusion genes in ESCs without FACT by ChIP-qPCR. Our ChIP-qPCR results revealed that H3K36me2 was enriched on MERVL-fused gene such as *Zfp809* (Figure 4(a)) but not on the control region (Figure 4(b)). However, this enrichment was decreased on MERVL-fusion genes in ESC without the *Ssrp1* expression (Figures 4(a) and 4(b)). Together, these suggest that the decreased enrichment of H3K36me2 on MERVL-fused genes may explain the activation of MERVL-fused genes after *Nsd2* downregulation in *Ssrp1*^−/−^ ESCs.

## 4. Discussion

In summary, we discovered that Nsd2 was a repressor of MERVL and MERVL-fused 2C genes, and the downregulation of *Nsd2* worked as a secondary regulatory route to activate MERVL after the loss of *Ssrp1*. It is interesting to see that only *Nsd2* (Figure 1(d)), but neither *Nsd1* nor *Nsd3*, is downregulated by the disruption of FACT function, given that all three Nsd genes participate in H3K36 methylation. Nsd2 is an important H3K36me2 methyltransferase [19, 22]. Loss of *Nsd2* mimics H3.3K36M mutation, but not *Nsd1* or *Setd2* mimics the effects of H3.3K36M on adipogenesis [23], implicating a unique role of Nsd2 among Nsd members in gene expression regulation. H3K6me2 was associated with both activation and repression of the gene expression [24]. It was recently reported that Nsd1/Nsd2-mediated intergenic H3K36me2 recruited Dnmt3a for DNA methylation [25, 26]. In yeast cells, H3K36me1/2/3 was also shown to repress cryptic transcription [27]. Moreover, H3K36me2 can recruit the Rpd3s histone deacetylase to repress spurious transcription [28]. These are consistent with our finding that H3K36me2 decreased on MERVL-fused genes after the loss of *Ssrp1* (Figures 4(a) and 4(b)), implying a potential repression role of H3K36me2.

Nsd2 is not only involved in gene transcription regulation. It participates in regulating genome stability and methylates non-histone proteins as well. Nsd2-mediated H3K36me2 promotes nonhomologous end-joining at unprotected telomeres and thereby enhances genomic instability caused by telomere dysfunction [29]. Human NSD2-mediated PTEN methylation regulates cell responses to DNA damage [30]. It is recently discovered that DNA damage is induced by the depletion of MERVL activator Zscan4 [3, 31]. Responses of ATR and CHK1 to replication stresses activate *Zscan4* and MERVL [20], implying that DNA damage-induced replication stress and Zscan4 reciprocally regulate each other. It will be interesting to investigate whether Nsd2 is involved DNA damage repair and its relationship with Zscan4 in the future.

## 5. Conclusion

In conclusion, we found that *Nsd2*, as a downstream gene of FACT, repressed MERVL, without influencing ESC pluripotency. The decreased *Nsd2* in *Ssrp1*^−/−^ ESCs was accompanied by reduced H3K36me2 on MERVL-fused genes while overexpression of *Nsd2* partially rescued the expression of MERVL. These findings establish Nsd2 as an important repressor of MERVL in ESCs and during the loss of FACT function.

## Figures and Tables

**Table 1:** Primers for qPCR analysis.

Gene	Forward	Reverse
*Nsd1*	TCCGGTGAATTTAGATGCCTCC	CGGTAACTGCATAGTACACCCAT
*Nsd2*	GGTGATCCTGGCACAGACAA	GAGCAGAGCCTGTGGACTTT
*Nsd3*	CCGAGGTTGTGCCAAAGAAG	ACGGAGCTGTCACTGAATCTG
MERVL	AAGAGCCAAGACCTGCTGAG	TCCTCGTTTCTGCAACTGGT
LINE1	GGACCAGAAAAGAAATTCCTCCCG	CTCTTCTGGCTTTCATAGTCTCTGG
SINEB1	GTGGCGCACGCCTTTAATC	GACAGGGTTTCTCTGTGTAG
*Oct4*	GTGGAAAGCAACTCAGAGG	GGTTCCACCTTCTCCAACT
*Sox2*	GCGGAGTGGAAACTTTTGTCC	CGGGAAGCGTGTACTTATCCTT
*Nanog*	TTGCTTACAAGGGTCTGCTACT	ACTGGTAGAAGAATCAGGGCT
*Zscan4*	GAGATTCATGGAGAGTCTGACTGATGAGTG	GCTGTTGTTTCAAAAGCTTGATGACTTC
*Dux*	CCCAGCGACTCAAACTCCTTC	GGACTTCGTCCAGCAGTTGAT
ChIP Control	GATTAGCAGCTCCACAGGA	TGGACAATGTGGCCTGTTTA
*Zfp809* ChIP	AAGCTGGCTGACTGTAGTGG	GTGAGCCTTCCAATTCCGGA
*Foxa2*	CCCTACGCCAACATGAACTCG	GTTCTGCCGGTAGAAAGGGA
*Sox17*	GATGCGGGATACGCCAGTG	CCACCACCTCGCCTTTCAC
*Gata4*	CCCTACCCAGCCTACATGG	ACATATCGAGATTGGGGTGTCT
*Nkx2.5*	GACAAAGCCGAGACGGATGG	CTGTCGCTTGCACTTGTAGC
*Msx1*	TGCTGCTATGACTTCTTTGCC	GCTTCCTGTGATCGGCCAT
*Pax6*	TACCAGTGTCTACCAGCCAAT	TGCACGAGTATGAGGAGGTCT
*Foxd3*	GAGTTCATCAGCAACCGTTTTC	CGAAGCTCTGCATCATCAGC
*Gata3*	CTCGGCCATTCGTACATGGAA	GGATACCTCTGCACCGTAGC

## Data Availability

Published ChIP-seq data we analyzed are GSE141791 for Ssrp1 [16], GSE117155 for H3K36me2 [32], GSE110321 for H3K36me3 [33], and GSE90893 for H3K4me3 [34].
